# Jabuticaba (*Myrciaria cauliflora*) Modulates Intestinal Inflammation, Liver Homeostasis, and Brain Gene Expression Along the Gut–Liver–Brain Axis in a DSS-Induced In Vivo Model

**DOI:** 10.3390/nu18060903

**Published:** 2026-03-12

**Authors:** Stephanie Michelin Santana Pereira, Vinícius Parzanini Brilhante de São José, Melissa Y. Huang, Lívya Alves Oliveira, Kelly Aparecida Dias, Júlia D’Almeida Francisquini, Italo Tuler Perrone, Ceres Mattos Della Lucia, Elad Tako

**Affiliations:** 1 Department of Food Science, Cornell University, Stocking Hall, Ithaca, NY 14850, USA; ss4442@cornell.edu (S.M.S.P.); vps24@cornell.edu (V.P.B.d.S.J.); mh2284@cornell.edu (M.Y.H.); 2 Department of Nutrition and Health, Universidade Federal de Viçosa, Viçosa 36570-900, MG, Brazil; livya.oliveira@ufv.br (L.A.O.); kelly.dias@ufv.br (K.A.D.); cmdellalucia@ufv.br (C.M.D.L.); 3 Department of Pharmaceutical Science, Universidade Federal de Juiz de Fora, Juiz de Fora 36036-900, MG, Brazil; juliafrancisquininutri@gmail.com (J.D.F.); italotulerperrone@gmail.com (I.T.P.)

**Keywords:** polyphenol, intestinal barrier, hepatic health, neurotrophic signaling, cecal microbiota, fruit, microencapsulation

## Abstract

**Background/Objectives:** Dextran sulfate sodium (DSS) is widely used to induce intestinal injury, reducing intestinal barrier integrity and thus contributing to systemic inflammation and oxidative stress, which may affect liver homeostasis and central nervous system function. In this context, the intake of phenolic compounds and anthocyanins from fruits such as jabuticaba has gained attention due to their antioxidant and anti-inflammatory properties. This study evaluated the effects of jabuticaba in the form of freeze-dried whole fruit, freeze-dried peel, and microencapsulated peel extract on DSS-induced damage to the gut–liver–brain axis in an in ovo model. **Methods:** Fertile eggs were assigned to five groups: water, DSS, DSS plus whole jabuticaba (WJ), DSS plus jabuticaba peel (JP), and DSS plus microencapsulated jabuticaba peel (JM). Duodenal, colon, and liver gene expressions; and histomorphometry, cecal microbiota, and brain gene expressions were evaluated at hatch. **Results:** DSS administration increased NF-κB expression and reduced MUC-2 in the duodenum, induced colonic inflammation, altered cecal microbiota, and caused hepatic oxidative stress, evidenced by elevated iNOS and enlarged fat globules, while reducing brain BDNF levels. Jabuticaba treatments mitigated intestinal, hepatic, and neural damage by reducing inflammatory markers; enhancing MUC-2, ZO-2, JAM-2, and claudin-1 expression; increasing villus area and goblet cell numbers; normalizing CAT and SOD activities in the liver; decreasing COX-2; increasing dopamine; and restoring BDNF in the brain. **Conclusions:** This study demonstrates that jabuticaba exerts protective effects along the gut–liver–brain axis, highlighting its potential as a functional food to support intestinal, hepatic, and brain health.

## 1. Introduction

The intestinal barrier consists of multiple layers that include various cell types, as well as physical–chemical and immunological components. Decreased number of goblet cells, reduced mucus thickness, and the downregulation of tight junction proteins contribute to intestinal barrier dysfunction, which can lead to chronic inflammation and ultimately to the development of inflammatory bowel disease (IBD). In addition, microbial products, endotoxins, and inflammatory signals can translocate to the portal vein, where they modulate hepatic inflammatory and metabolic responses involved in the pathophysiology of liver diseases [1,2,3,4]. Also, these signals can reach the central nervous system through circulatory routes or via vagal pathways, where they modulate neuroinflammatory processes, neurotransmitter balance, and neuronal function, contributing to the pathophysiology of neurological and behavioral disorders [5,6,7,8].

Among the various chemically induced IBD models, the model induced by dextran sulfate sodium (DSS) has been widely utilized due to its simplicity and its similar effects as human IBD. Studies have shown that DSS administration induced clinical, macroscopic, and histological features associated with enteritis, such as intestinal injury, reduced villus height, and changes in goblet cell density. Moreover, DSS increases intestinal permeability and induces the production of pro-inflammatory cytokines. Consequently, these alterations affect liver health, worsening hepatic inflammation and fibrosis, and increase circulating endotoxins, which have been associated with microglial activation, blood–brain barrier (BBB) dysfunction, and impaired neuronal signaling [9,10,11,12,13,14].

Fruits from the *Myrciaria* genus, such as jabuticaba, a Brazilian berry, are notable sources of phenolic compounds, particularly anthocyanins, ellagic acid, and other bioactive polyphenols, with well-established antioxidant, anti-inflammatory, and neuroprotective properties in preclinical models [15,16]. Evidence suggests that these compounds enhance intestinal barrier function by reducing inflammatory cytokine expression, modulating gut microbiota composition, and supporting epithelial morphology [17,18,19]. As for hepatic functions, phenolic extracts of jabuticaba have demonstrated hepatoprotective effects in experimental models, reducing markers of oxidative stress, inflammation, and histopathological alterations, and thus suggesting their potential to modulate hepatic responses to signals originating from the gut [20,21,22]. In the brain, these polyphenols and metabolites may influence central nervous system health by attenuating neuroinflammation, improving redox balance, and modulating pathways involved in synaptic plasticity and neurotransmission [23,24].

However, the stability and bioavailability of anthocyanins and other polyphenols remain major technical challenges. Drying techniques such as freeze-drying and microencapsulation methods such as spray-drying have been used to protect these compounds against adverse conditions (pH, temperature, and gastric juice) and to improve their release and bioaccessibility [25,26,27]. Therefore, comparing different application forms (freeze-dried whole fruit, freeze-dried peel, and microencapsulated peel) allows investigators to determine whether processing methods alter the biological effects on the intestine, liver, and brain.

Moreover, the in ovo model using intra-amniotic administration combined with DSS has been successfully adapted to investigate mechanisms of intestinal inflammation and to screen nutritional and phytotherapeutic interventions in a highly controlled and reproducible developmental system. This approach allows for the direct exposure of the intestinal epithelium to bioactive compounds before hatching, enabling the assessment of early intestinal responses, barrier function, immune activation, and systemic outcomes shortly. Thus, this well-established in vivo model represents a robust and ethically favorable platform to explore gut–liver–brain axis interactions and generate mechanistic insights with potential relevance to human intestinal health [28,29,30,31,32,33].

Based on these considerations, the present study investigated the effects of jabuticaba extracts administered with DSS in an in ovo (*Gallus gallus*) model, with a specific focus on the gut–liver–brain axis. We hypothesized that treatments containing jabuticaba, principally microencapsulated peel, would mitigate DSS-induced intestinal injury, preserve barrier integrity, reduce the activation of inflammatory responses and fat accumulation in hepatic tissue, and attenuate neuroinflammatory pathways potentially triggered by gut-derived inflammatory and metabolic disturbances.

## 2. Materials and Methods

### 2.1. Raw Material

Fruits of *Myrciaria cauliflora* were collected during the harvest season, from producers located in the rural area of Jequeri, Minas Gerais, Brazil. The jabuticabas were sanitized and manually separated into two distinct portions: whole fruit and peel. These portions were then freeze-dried and subsequently ground in a knife mill until a homogeneous powder was obtained. To encapsulate the anthocyanins present in jabuticaba, an extract was obtained from the previously freeze-dried peels.

The peels were submitted to hydroethanolic extraction (50% *v*/*v*) and agitated in a metabolic bath at 22 ± 2 °C, 180 rpm for 10 min (Marconi^®^ model MA-093, Marconi Equipamentos para Laboratório Ltda, Piracicaba, SP, Brazil). The extract was then centrifuged (Nüve^®^ centrifuge, NF 1200/1200R, Nüve Sanayi Malzemeleri İmalat ve Ticaret A.Ş., Ankara, Turkey) at 3500 rpm for 15 min, and the supernatant was vacuum-filtered and concentrated by evaporation under reduced pressure at 40 °C for 20 min using a rotary evaporator coupled to a vacuum pump [34]. The extracts were produced at the Vitamin Analysis Laboratory, Universidade Federal de Viçosa, Minas Gerais, Brazil. Subsequently, the extract was dried using a spray dryer (Labmaq^®^, model MSDi 1.0, Labmaq do Brasil Ltda, Ribeirão Preto, SP, Brazil) equipped with a 1 mm spray nozzle, using maltodextrin as an encapsulating agent. Air and product flow rates, as well as inlet air temperature, were defined according to the methodology proposed by Perrone et al. (2013) [35]. The drying flow rate was set at 1 kg/h, with the sample dried at 45 °C. This process was performed at the Universidade Federal de Juiz de Fora, Minas Gerais, Brazil.

### 2.2. Biological Assay

Before the experiment starts, the freeze-dried whole jabuticaba, freeze-dried jabuticaba peel, and microencapsulated jabuticaba peel were diluted in ultrapure water (18 MΩ H_2_O) in three different dilution concentrations (1%, 5%, and 10%) to determine the concentration that would be administered in order to maintain an osmolarity value (OSM) lower than 320 Osm and, thus, ensure that the chicken embryos were not dehydrated after injection of the solution [31]. The chosen dose was 5%, since the intention was to use the same concentration among the extracts, and the jabuticaba peel extract presented an osmolarity of 320 mOsm at a concentration of 10% (Table 1).

The following analyses were performed on the solution used for in ovo injection: total phenolic content by Singleton et al. (1999) [36], monomeric anthocyanins by Giusti and Wrolstad (2003) [37], and antioxidant capacity using the 1,1-diphenyl-2-picrylhydrazyl (DPPH) radical scavenging assay by Bloor (2001) [38], the Trolox equivalent antioxidant capacity (TEAC) assay by Pavan et al. (2014) [39], and the ferric-reducing antioxidant power (FRAP) assay by Oyaizu (1986) [40].

Fertile eggs (n = 111) were obtained from a commercial hatchery (Cobb, Siloam Springs, AR, USA) and incubated under optimal conditions (37 ± 2 °C and 89.6 ± 2% humidity) in the incubator at the Cornell University Department of Animal Science poultry farm. On the 14th day of embryonic incubation, the eggs were screened using a dedicated light to discard those with infertile, cracked, contaminated, or prematurely dead embryos, leaving 100 viable eggs.

**Figure 1:**
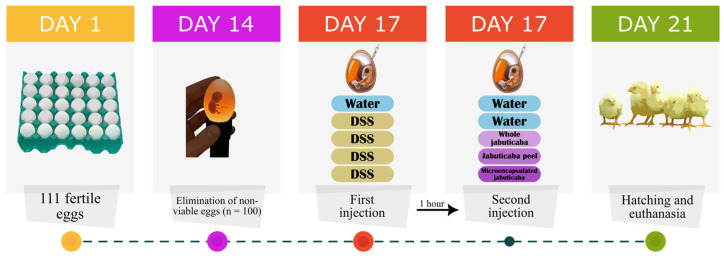
Experimental design.

On the 17th day of incubation, the eggs were weighed and randomly allocated into 5 groups (n = 20/group) with similar weight distribution. The eggs were distributed into the following groups: (1) water (18 MΩ H_2_O); (2) DSS (1.5%); (3) whole jabuticaba (5%) + DSS (1.5%); (4) jabuticaba peel (5%) + DSS (1.5%); and (5) jabuticaba microencapsulated (5%) + DSS (1.5%; Figure 1).

After distributing the eggs, the amniotic fluid was identified via candling, and the injection site was marked and disinfected with 70% ethanol. Then, using a 19 mm needle inserted vertically, the eggs in group 1 were injected with 1 mL of water, and groups 2 to 5 were injected with 1 mL of DSS. One hour after the first application, the eggs in groups 1 and 2 were injected with another 1 mL of water, and groups 3 to 5 were injected with 1 mL of the respective extracts. After each injection, the eggs were disinfected with 70% ethanol and sealed with cellophane tape. After the second injection, they were placed in incubation baskets so that each treatment was equally represented in each incubator location. On the 21st day, only 8 eggs per group hatched. Immediately after hatching (day 21st), the animals were weighed and euthanized by exposure to CO_2_. The brain, liver, duodenum, colon, and cecal content were collected.

### 2.3. Analysis

#### 2.3.1. Real-Time Polymerase Chain Reaction (RT-qPCR)

For the evaluation of gene expression in duodenum, colon, liver, and brain tissues, total RNA was isolated using TRIzol reagent (QIAzol Lysis Reagent, Qiagen^®^, Germantown, MD, USA). The extracted RNA was subsequently reverse-transcribed into complementary DNA (cDNA) with the High-Capacity cDNA Reverse Transcription Kit (Applied Biosystems^®^, Foster City, CA, USA), following the manufacturer’s instructions, using a C1000 Touch Thermal Cycler (Bio-Rad Laboratories^®^, Hercules, CA, USA) [41].

Quantitative real-time PCR (RT-qPCR) analyses were carried out using PerfeCTa^®^ SYBR Green FastMix^®^ (Quantabio, Beverly, MA, USA) on a CFX96 Touch Real-Time PCR Detection System (Bio-Rad Laboratories^®^, Hercules, CA, USA). Primer sets were designed to amplify genes associated with inflammatory and barrier-related pathways in the intestine; nuclear factor kappa B (NF-κB), interleukin-1β (IL-1β), mucin-2 (MUC-2), junctional adhesion molecule 2 (JAM-2), zonula occludens-2 (ZO-2), claudin-1 (CLDN-1), inducible nitric oxide synthase (iNOS), catalase (CAT), superoxide dismutase 1 (SOD-1), and glutathione peroxidase (GSH-Px) on the liver; and cyclooxygenase-2 (COX-2), dopamine, and brain-derived neurotrophic factor (BDNF) in the brain. Gene expression was quantified using β-actin as the endogenous control, and results were calculated according to the 2^−ΔΔCt^ method (Table 2) [41].

#### 2.3.2. Histomorphometry

For histomorphometry analysis, liver and duodenum samples were collected immediately after euthanasia and fixed in 10% neutral buffered formalin for 72 h prior to processing. The formalin-fixed tissues were transversely sectioned to obtain samples suitable for morphological analysis. Sections were then dehydrated, cleared, and embedded in paraffin [42]. Serial sections of 5 µm thickness were prepared, mounted on glass slides, deparaffinized in xylene, and rehydrated through a graded ethanol series. Duodenal samples were stained using Alcian Blue–Periodic Acid–Schiff (AB-PAS), while liver sections were stained with hematoxylin and eosin.

Images were captured with an Olympus BX43 portable microscope fitted with a 2.8-megapixel DP22 digital camera (Olympus Life Science Solutions, Tokyo, Japan) using a 10× objective. Morphometric analysis of the duodenum included measurements of villi (length, width, and surface area), crypts (length and width), goblet cell number and area, and the longitudinal and circular thickness of the muscular layer. Villi surface area was estimated by modeling each villus as a cylinder, using the measured height and the average width of three villi [28]. For colon, only the villus measurements were not performed.

For liver samples, cytoplasmic and lipid droplet areas, inflammatory regions, and hepatic nuclei were quantified using tissue point counting with a 266-point grid, totaling 1064 points per animal [43]. All morphometric analyses were conducted using Image-Pro-Plus^®^ software, version 4.5.0 (Media Cybernetics, Rockville, MD, USA).

#### 2.3.3. Cecal Bacterial

Microbial DNA was isolated from frozen cecal contents. While still frozen, the material was finely fragmented and transferred to tubes containing glass beads and PBS (pH 7.4). Samples were vortexed for 3 min to disrupt the material and centrifuged at 1000× *g* for 3 min. The liquid phase was transferred to fresh tubes, avoiding residual solids, and subjected to an additional centrifugation step at 4000× *g* for 10 min at 4 °C. The pellet obtained was washed with PBS (pH 6.8) and centrifuged again under the same conditions. Subsequently, the pellet was resuspended in 50 mM EDTA and incubated with lysozyme at 37 °C for 1 h to support enzymatic digestion of bacterial cell walls.

After centrifugation at 16,300× *g* for 2 min and removal of the supernatant, chemical lysis was performed using the Nuclei Lysis Solution from the Promega Wizard DNA Purification Kit (Promega, Madison, WI, USA), followed by sequential incubations at 80 °C and 37 °C. Proteins were precipitated with Protein Precipitation Solution and removed by centrifugation. DNA was recovered by adding isopropanol to the cleared supernatant, and the resulting pellet was washed with 70% ethanol, air-dried, and dissolved in DNA Rehydration Solution. Samples were stored at 4 °C overnight, and DNA concentration and purity were evaluated using 260/280 and 260/230 absorbance ratios measured with a NanoDrop 2000 spectrophotometer (Thermo Fisher Scientific^®^, Waltham, MA, USA) [28,29].

Amplification of 16S rRNA gene fragments was performed using microbial DNA isolated from the cecal samples. Each 25 µL PCR reaction was prepared with GoTaq Green Master Mix (Promega), nuclease-free water, genus-specific primers, and template DNA. DNA input was standardized to approximately 1000–1500 ng per reaction based on NanoDrop measurements. Reactions were run on a Bio-Rad C1000 thermocycler (Bio-Rad Laboratories) for 40 cycles, using annealing temperatures between 50 and 60 °C according to primer requirements. A no-template control was included in each run to detect potential contamination.

PCR products were analyzed via agarose gel electrophoresis. Gels were prepared in 1× TAE buffer with GelRed stain, poured into trays, allowed to solidify, and then submerged in 1× TAE. A 12 µL aliquot of each reaction was loaded into wells alongside a DNA ladder. Electrophoresis was conducted at 100 V for roughly 45 min under light-protected conditions. The gels were then imaged on a gel documentation system, and band intensities were quantified as signal per unit area (Int/mm^2^) [28,29].

### 2.4. Statistical Analysis

Data normality was assessed using the Shapiro–Wilk test. The *t*-test was used to assess differences within the DSS groups (DSS × WJ + DSS; DSS × JP + DSS; DSS × JM + DSS). Intergroup differences (water, DSS, WJ + DSS, JP + DSS and JM + DSS) were submitted to analysis of variance (ANOVA), followed by Tukey’s mean test. A *p*-value < 0.05 was adopted. The results are expressed as mean and standard deviation (mean ± SD). Statistical analysis and graph construction were performed using GraphPad Prism^®^ software, version 10.1.2 (GraphPad Prism Inc., La Jolla, CA, USA).

### 2.5. Ethical Aspects

The Cornell University Institutional Animal Care and Use Committee approved this project under the protocol code 2020-0077.

## 3. Results

Chemical analysis determined that freeze-dried jabuticaba peel (JP) contained the highest levels of total phenolics and monomeric anthocyanins, followed by freeze-dried whole jabuticaba (WJ), while the microencapsulated extract (JM) displayed substantially lower concentrations. Antioxidant capacity measured by DPPH, ABTS, and FRAP assays followed the same pattern, with JP exhibiting the strongest activity, WJ intermediate values, and JM the lowest (Table 3).

**Figure 2:**
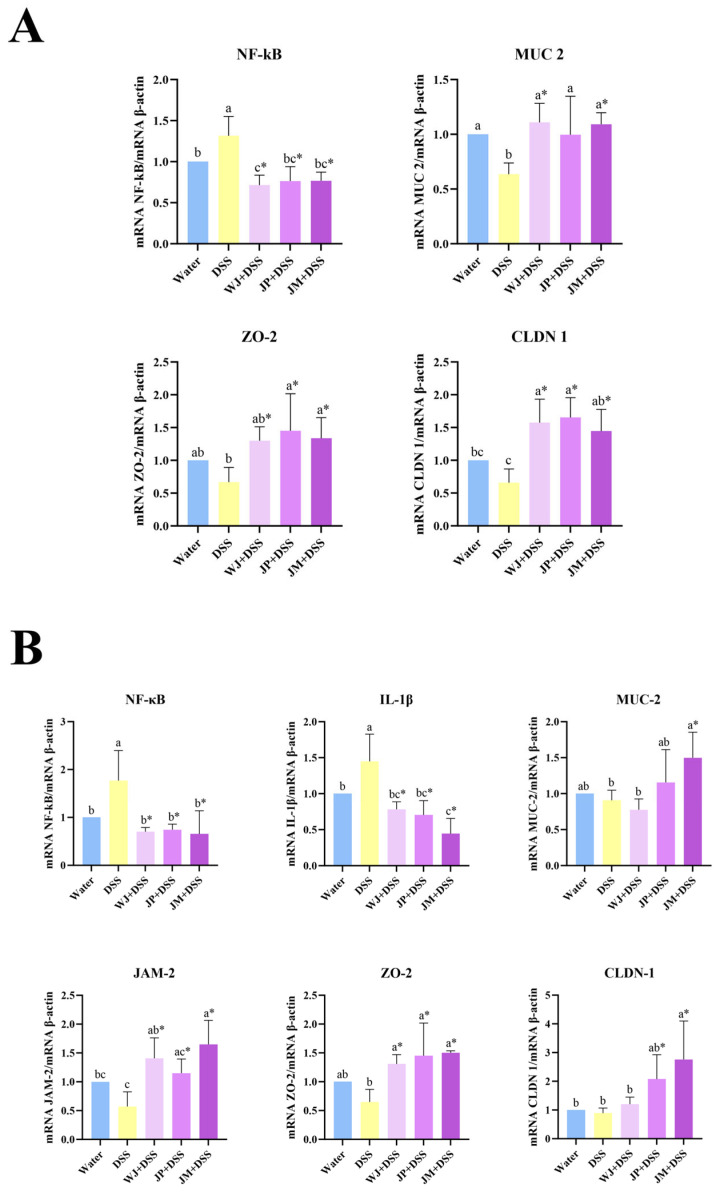
Gene expression profile in the duodenum and colon. (**A**) Duodenum and (**B**) colon. NF-κB, nuclear factor kappa B; IL-1β, interleukin-1β; MUC-2, mucin 2; JAM-2, junctional adhesion molecule 2; ZO-2, zona occludens-2; CLDN-1, Claudin 1; water, 18 MΩ H_2_O; DSS, dextran sulfate sodium 1.5%; WJ + DSS, whole jabuticaba (5%) + DSS (1.5%); JP + DSS, jabuticaba peel (5%) + DSS (1.5%); JM + DSS, Jabuticaba microencapsulated (5%) + DSS (1.5%). n = 5/group. * Indicates significant differences between the DSS groups (DSS × WJ + DSS; DSS × JP + DSS; DSS × JM + DSS), according to the *t*-test (*p* < 0.05). Different lowercase letters (a–c) indicate significant differences within groups, according to ANOVA, followed by Tukey’s test, at 5% probability. Data expressed as mean ± standard deviation.

Upon analyzing intestinal gene expression, in the duodenum, animals exposed to DSS presented a significant upregulation of NF-κB (*p* = 0.0362) and reduction in MUC-2 (*p* = 0.0277) expression compared with the water group. Treatment with whole jabuticaba, jabuticaba peel, or microencapsulated jabuticaba significantly reduced NF-κB expression (*p* < 0.0001, *p* = 0.0002, and *p* = 0.0003, respectively) and increased MUC-2 expression (*p* = 0.0032, *p* = 0.0446, and *p* = 0.0046, respectively) relative to the DSS group. Regarding markers of epithelial barrier integrity, DSS tended to reduce the expression of tight junction-related genes. All jabuticaba-treated groups presented significantly higher expression of claudin-1 expression (*p* = 0.0024, *p* = 0.0010, and *p* = 0.0092, respectively), and the JP + DSS and JM + DSS groups presented an increase in ZO-2 compared to DSS alone (*p* = 0.0150, and *p* = 0.0447, respectively; Figure 2A).

In the colon, DSS administration markedly increased the expression of both NF-κB (*p* = 0.0143) and IL-1β (*p* = 0.0183) compared to the water group. Supplementation with jabuticaba in all tested forms significantly reduced the expression of these pro-inflammatory markers compared to DSS alone. In relation to the expression of barrier-associated genes, all jabuticaba interventions significantly upregulated ZO-2 relative to the DSS group (*p* = 0.0103, *p* = 0.0017, and *p* = 0.0009, respectively), and WJ and JM increased the JAM-2 expression (*p* = 0.0035 and *p* = 0.0002, respectively). Only JM intervention increased the claudin-1 expression compared to DSS alone (*p* = 0.0066).

**Figure 3:**
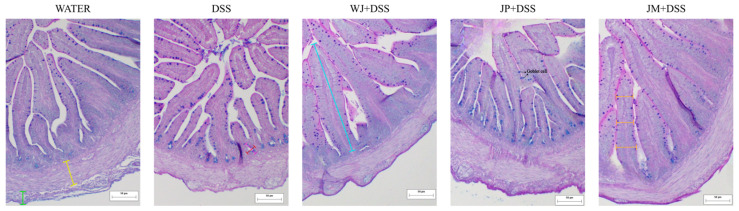
Representative duodenum photomicrographs stained with Alcian Blue–Periodic Acid–Schiff (AB-PAS). Water, 18 MΩ H_2_O; DSS, dextran sulfate sodium 1.5%; WJ + DSS, whole jabuticaba (5%) + DSS (1.5%); JP + DSS, jabuticaba peel (5%) + DSS (1.5%); JM + DSS, jabuticaba microencapsulated (5%) + DSS (1.5%). Blue line, villi length; orange line, villi width; green line, longitudinal muscle layer thickness; yellow line, circular muscle layer thickness; pink line, crypt length; red line, crypt depth; goblet cell is indicated in the image.

The thickness of the duodenal longitudinal muscle layer was significantly reduced by DSS compared with the water group (*p* = 0.0107), while the jabuticaba treatments presented distinct effects; specifically, JP + DSS exhibited a significant increase in duodenal longitudinal muscle layer relative to DSS (*p* = 0.0022; Table 4 and Figure 3). Villus surface area, however, was affected. Specifically, all jabuticaba treatments resulted in a significant increase in villus surface area, relative to the DSS group (*p* <0.0001). Crypt length was greater in the DSS group compared with water (*p* = 0.0349), reflecting inflammation-induced hyperplasia. Administration of jabuticaba peel attenuated this effect, as JP + DSS and JM + DSS presented significantly reduced crypt length compared with DSS (*p* < 0.0001 and *p* = 0.0170, respectively; Table 4 and Figure 3).

For goblet cells per villus, all jabuticaba treatments significantly increased goblet cells number compared with DSS (*p* = 0.0012, *p* < 0.0001, and *p* = 0.0008, respectively). Regarding goblet cell number per crypt, WJ and JM presented significant increases relative to DSS (*p* = 0.0005 and *p* = 0.0058, respectively), indicating a restorative effect. For goblet cell area, only JM presented a significantly greater area than DSS (*p* = 0.0307; Table 4 and Figure 3).

**Figure 4:**
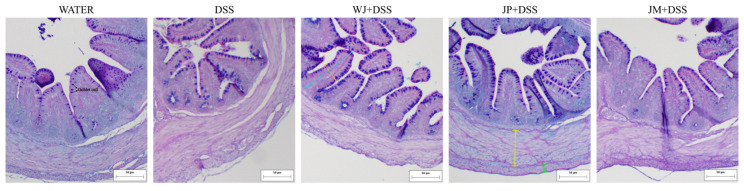
Representative photomicrographs of colon sections stained with Alcian Blue–Periodic Acid–Schiff (AB–PAS). Water, 18 MΩ H_2_O; DSS, dextran sulfate sodium 1.5%; WJ + DSS, whole jabuticaba (5%) + DSS (1.5%); JP + DSS, jabuticaba peel (5%) + DSS (1.5%); JM + DSS, jabuticaba microencapsulated (5%) + DSS (1.5%). Blue line, crypt depth; orange line, crypt width; green line, longitudinal thickness; yellow line, circular thickness; goblet cell is indicated in the image.

In the colon, DSS administration resulted in increases in both the longitudinal (*p* < 0.0001) and circular (*p* = 0.0005) intestinal muscle layers compared to the healthy control group. All three jabuticaba treatments were effective in reducing the thickness of the longitudinal muscle layer relative to the DSS group (*p* < 0.0001), with a more pronounced reduction observed in the JP + DSS and JM + DSS groups. In addition, the JP + DSS and JM + DSS groups also decreased the thickness of the circular muscle layer compared to DSS alone (*p* < 0.0001; Table 5 and Figure 4).

Regarding colon crypts, DSS exposure led to a reduced crypt depth compared to the water group (*p* = 0.0007). All jabuticaba treatments increased crypt depth compared to the DSS group (*p* < 0.0001, *p* = 0.0021, and *p* = 0.0002, respectively); however, only the WJ + DSS and JM + DSS groups were effective in increasing crypt width relative to DSS (*p* = 0.0007 and *p* = 0.0157, respectively; Table 5 and Figure 4). The jabuticaba treatments also present an increase in number of goblet cells per crypt (*p* < 0.0001, *p* = 0.0060, and *p* = 0.0038, respectively) and goblet cell area (*p* = 0.0001, *p* = 0.0016, and *p* = 0.0004, respectively) when compared to the DSS group (Table 5 and Figure 4).

**Figure 5:**
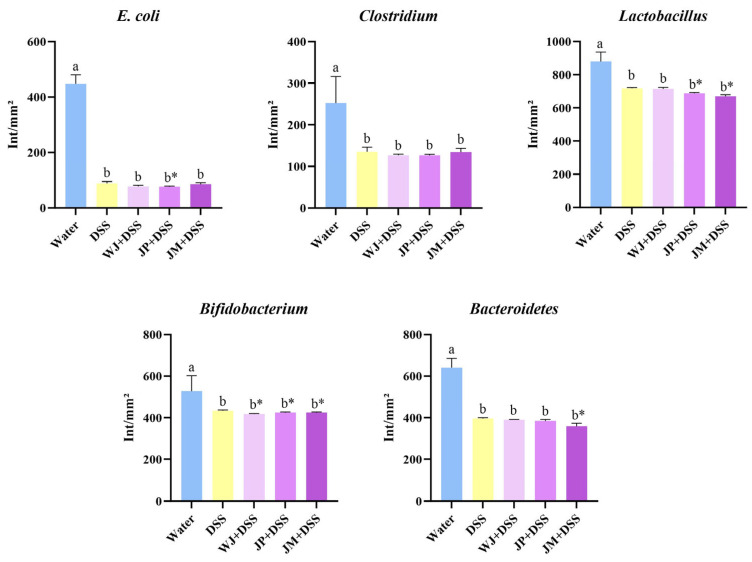
Cecal microbiota composition. Water, 18 MΩ H_2_O; DSS, dextran sulfate sodium 1.5%; WJ + DSS, whole jabuticaba (5%) + DSS (1.5%); JP + DSS, jabuticaba peel (5%) + DSS (1.5%); JM + DSS, jabuticaba microencapsulated (5%) + DSS (1.5%). n = 5/group. * Indicates significant differences between the DSS groups (DSS × WJ + DSS; DSS × JP + DSS; DSS × JM + DSS), according to the *t*-test (*p* < 0.05). Different lowercase letters (a,b) indicate significant differences within groups, according to ANOVA, followed by Tukey’s test, at 5% probability. Data expressed as mean ± standard deviation.

Analysis of the cecal microbiota showed that DSS administration reduced the abundance of *Escherichia coli*, *Clostridium*, *Lactobacillus*, *Bifidobacterium*, and *Bacteroidetes* compared with the healthy control group (*p* < 0.0001, *p* < 0.0001, *p* = 0.0096, *p* = 0.0163, and *p* < 0.0001, respectively). With respect to *E. coli*, the JP + DSS group exhibited an additional reduction relative to DSS (*p* = 0.0421). In the case of *Lactobacillus*, the JP and JM treatments showed further reductions when compared with DSS (*p* = 0.0181 and *p* = 0.0185, respectively). Results for *Bifidobacterium* indicated that jabuticaba-treated groups showed significant decrease when directly compared with DSS (*p* = 0.0058, *p* = 0.0361, and *p* = 0.0406, respectively; Figure 5). Analysis of *Bacteroidetes* demonstrated that the JM + DSS group exhibited an additional reduction relative to DSS (*p* = 0.0458; Figure 5).

**Figure 6:**
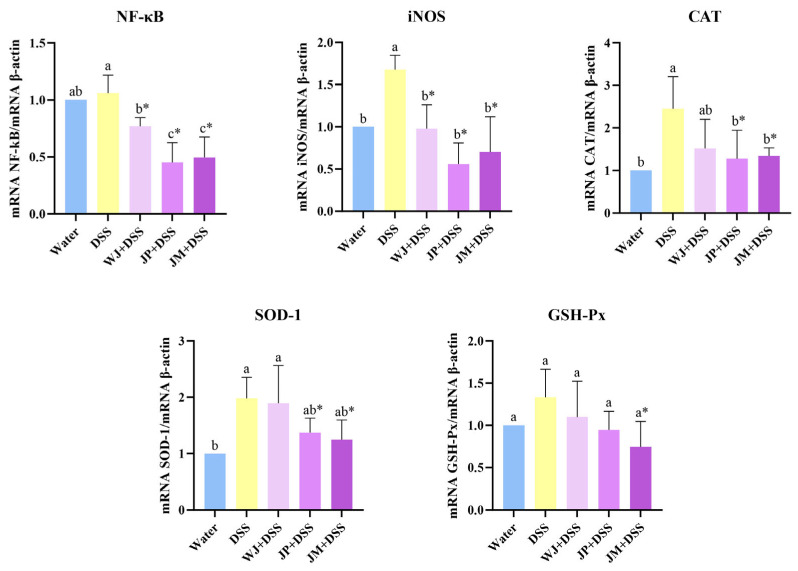
Gene expressions of NF-κB, iNOS, CAT, SOD-1, and GSH-Px in liver. NF-κB, nuclear factor kappa B; iNOS, inducible nitric oxide synthase; CAT, catalase; SOD-1, superoxide dismutase 1; GSH-Px, glutathione peroxidase. Water, 18 MΩ H_2_O; DSS, dextran sulfate sodium 1.5%; WJ + DSS, whole jabuticaba (5%) + DSS (1.5%); JP + DSS, jabuticaba peel (5%) + DSS (1.5%); JM + DSS, jabuticaba microencapsulated (5%) + DSS (1.5%). n = 5/group. * Indicates significant differences between the DSS groups (DSS × WJ + DSS; DSS × JP + DSS; DSS × JM + DSS), according to the *t*-test (*p* < 0.05). Different lowercase letters (a–c) indicate significant differences within groups, according to ANOVA, followed by Tukey’s test, at 5% probability. Data expressed as mean ± standard deviation.

As for hepatic gene expressions, all forms of jabuticaba reduced NF-κB (*p* = 0.0116, *p* = 0.0014, and *p* = 0.0078, respectively), with JP and JM appearing to be the most effective. On the other hand, DSS significantly increased iNOS (*p* = 0.0126) and CAT (*p* = 0.0019) expressions compared with the water group. All jabuticaba-treated groups presented a significant reduction in iNOS (*p* = 0.0134, *p* = 0.0031, and *p* = 0.0134, respectively) compared with DSS alone, and the JP and JM treatments reduced the CAT expression compared with DSS (*p* = 0.0319 and *p* = 0.0134, respectively; Figure 6). DSS increased SOD-1 expression relative to the water group (*p* = 0.0060), while the JP and JM treatments presented significant reductions compared with DSS (*p* = 0.0370 and *p* = 0.0290, respectively). For GSH-Px, only the JM treatment presented significantly reduced GSH-Px expression relative to DSS (*p* = 0.0383; Figure 6).

**Figure 7:**
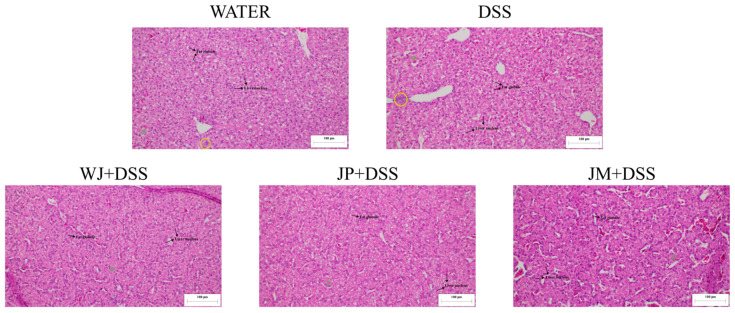
Representative liver photomicrographs stained with hematoxylin and eosin. Water, 18 MΩ H_2_O; DSS, dextran sulfate sodium 1.5%; WJ + DSS, whole jabuticaba (5%) + DSS (1.5%); JP + DSS, jabuticaba peel (5%) + DSS (1.5%); JM + DSS, jabuticaba microencapsulated (5%) + DSS (1.5%). Yellow circle, inflammatory infiltrates; green circle, cytoplasm. Fat globules and liver nucleus are indicated in the images.

The morphometric analysis of liver tissue presented that the JM + DSS group exhibited a significant increase in nuclear area compared with the DSS group (*p* = 0.0021). Regarding the hepatocyte cytoplasm, DSS induced a significant reduction in cytoplasm compared with the water group (*p* < 0.0001), indicating structural impairment. All jabuticaba-treated groups presented a significantly increased cytoplasmic area compared with DSS (*p* < 0.0001; Table 6 and Figure 7). Quantification of fat globules revealed that DSS significantly increased hepatic lipid deposition relative to the water group (*p* < 0.0001). All three jabuticaba treatments significantly reduced these lipid accumulations (*p* < 0.0001). Regarding inflammatory infiltrates, the jabuticaba treatments markedly reduced these infiltrates relative to the DSS group (*p* < 0.0001; Table 6 and Figure 7).

**Figure 8:**
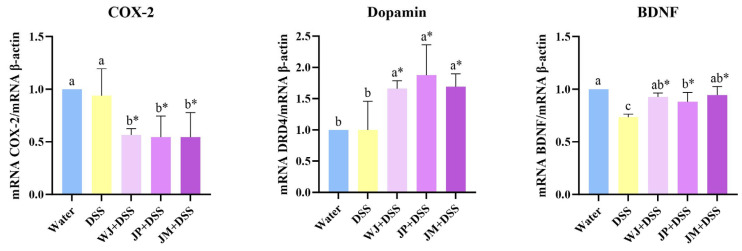
Quantitative analysis of brain mRNA gene expression. COX-2, cyclooxygenase-2; BDNF, brain-derived neurotrophic factor; water, 18 MΩ H_2_O; DSS, dextran sulfate sodium 1.5%; WJ + DSS, whole jabuticaba (5%) + DSS (1.5%); JP + DSS, jabuticaba peel (5%) + DSS (1.5%); JM + DSS, jabuticaba microencapsulated (5%) + DSS (1.5%). n = 5/group. * Indicates significant differences between the DSS groups (DSS × WJ + DSS; DSS × JP + DSS; DSS × JM + DSS), according to the *t*-test (*p* < 0.05). Different lowercase letters (a–c) indicate significant differences within groups, according to ANOVA, followed by Tukey’s test, at 5% probability. Data expressed as mean ± standard deviation.

The WJ, JP, and JM treatments exhibited reduced COX-2 gene expression (*p* = 0.0153, *p* = 0.0361, and *p* = 0.0479, respectively) and increased dopamine gene expression (*p* = 0.0148, *p* = 0.0188, and *p* = 0.0154, respectively) compared with DSS alone. However, a reduction in BDNF gene expression was observed in the DSS group relative to the water group (*p* < 0.0001), and all three jabuticaba-treated groups presented an increased gene expression relative to the DSS group (*p* < 0.0001, *p* = 0.0085, and *p* = 0.0006, respectively; Figure 8).

## 4. Discussion

The present study demonstrated that different forms of jabuticaba, freeze-dried whole fruit, freeze-dried peel, and microencapsulated peel extract were able to attenuate DSS-induced alterations along the gut–liver–brain axis in the in ovo model, indicating that the bioactive compounds in jabuticaba exert important protective actions against inflammatory and oxidative injury. These findings support recent evidence showing that Brazilian berries, due to their high content of anthocyanins and ellagitannins, exert modulatory effects on intestinal barrier, hepatic homeostasis, and neuroplasticity, particularly under conditions of mucosal imbalance and increased intestinal permeability [44,45,46].

DSS administration effectively induced intestinal inflammation, as demonstrated by the marked upregulation of NF-κB, in the duodenum and colon, and IL-1β expression in the colon. Activation of NF-κB represents a central event in DSS-induced epithelial injury, driving the transcription of pro-inflammatory cytokines and perpetuating mucosal immune activation [47,48]. The concomitant increase in IL-1β further reflects inflammasome-related signaling and epithelial stress, which together contributes to the breakdown of epithelial homeostasis [49]. These molecular alterations were paralleled by significant histomorphometric changes, including a decreased villus surface area in the duodenum, reduced crypt depth, and decreased goblet cell numbers in the colon. Collectively, these findings confirm the establishment of a robust inflammatory phenotype and compromised mucosal architecture.

In parallel with inflammatory signaling, DSS exposure impaired markers associated with epithelial barrier integrity, notably reducing the expression of JAM-2 in the colon. Tight junction proteins are essential for maintaining paracellular permeability and preventing luminal antigens from accessing the lamina propria. Their downregulation suggests increased intestinal permeability, a hallmark of inflammatory bowel conditions and a critical initiating factor in gut-derived systemic signaling [50,51,52]. Interestingly, DSS did not significantly alter MUC-2, ZO-2, or CLDN-1 expression at the molecular level in the colon, despite the observed reduction in goblet cell number in the colon and MUC-2 in the duodenum.

This apparent discrepancy may reflect the acute nature of the model, in which transcriptional changes in mucin-related genes may occur later than structural and cellular changes. In fact, even when the mucus layer is thickened in response to inflammatory stimuli, many bacteria are still able to penetrate the mucus and reach the intestinal epithelium. This bacterial penetration may result from reduced glycosylation and sulfation of mucins, as well as increased acidification of sialic acid, which together weaken the protective function of the mucus barrier [53,54,55]. It is also important to recognize that the DSS exposure used in the in ovo model differs substantially from classical DSS-induced colitis models commonly employed in rodents. In the in ovo system, DSS exposure occurs during embryonic development and over a short experimental period, which primarily reflects acute epithelial responses rather than the chronic inflammatory processes typically observed in mammalian models of colitis.

Jabuticaba treatments markedly attenuated DSS-induced inflammatory signaling, reducing NF-κB, in the duodenum and colon, and IL-1β expression in the colon across all formulations tested. These effects are consistent with the well-documented anti-inflammatory properties of jabuticaba polyphenols, particularly anthocyanins and ellagic acid derivatives, which are known to interfere with NF-κB activation and cytokine production [17,19,56]. Beyond suppressing inflammation, jabuticaba treatments enhanced markers related to barrier integrity, increasing MUC-2 in the duodenum, JAM-2 in the colon, and ZO-2 and CLDN-1 gene expressions in the duodenum and colon relative to the DSS group. Also, jabuticaba causes improvements in structural markers in the duodenum, such as increased villus surface area and greater numbers of goblet cells per villi and crypt. These findings not only indicate that jabuticaba dampens inflammatory cascades but also reinforce that jabuticaba phenolic compounds act directly to restore the physical and functional integrity of the intestinal epithelium [17,18,57].

Although all forms produced significant benefits, some subtle differences were observed in the duodenum. The freeze-dried peel showed more pronounced improvements in intestinal muscular parameters and crypt hyperplasia, suggesting a more direct intestinal effect, likely due to the concentration of phenolics and fiber in this fraction. The literature highlights that the peel contains the majority of jabuticaba’s anthocyanins and ellagitannins, conferring greater bioactive potency [16,58,59]. On the other hand, the microencapsulated jabuticaba preparation stood out by increasing goblet cell area, suggesting superior modulation of mucus production. This is consistent with the fact that microencapsulation preserves the stability and integrity of phenolic compounds, preventing oxidative degradation and allowing for controlled release throughout the gastrointestinal tract [60,61]. Thus, microencapsulation may have contributed to greater biological availability of active compounds in the intestine, enhancing their effect on mucogenesis [44].

In the colon, the microencapsulated jabuticaba peel consistently showed the most pronounced effects on barrier-related outcomes, and it exhibited increased expression of MUC-2 and CLDN-1. These results highlight the importance of processing strategies in determining the biological efficacy of polyphenol-rich interventions. Microencapsulation likely enhanced the stability and bioaccessibility of jabuticaba anthocyanins and other phenolic compounds, protecting them from degradation during gastrointestinal transit and increasing their availability at the intestinal epithelium; however, this hypothesis was not directly evaluated in the present study, as no pharmacokinetic or metabolite analyses were performed [26,60,61].

Previous studies have shown that microencapsulation strategies can improve phenolic absorption and prolong their presence in the intestinal lumen, allowing these compounds to exert local effects on epithelial cells, including colonocytes, independently of extensive microbial biotransformation [26,60,62,63]. In this context, the beneficial effects observed in the present study may be partially attributed to direct interactions between bioaccessible phenolics and intestinal epithelial and immune cells, rather than being exclusively mediated by microbiota modulation [64,65]. Enhanced mucus production, evidenced by increased goblet cell number and area, is particularly relevant, as the mucus layer represents the first line of defense against luminal antigens, microbial products, and inflammatory stimuli. Together, these findings suggest that microencapsulated jabuticaba may reinforce intestinal barrier function through direct epithelial mechanisms.

Despite significant improvements in intestinal inflammation and barrier-related parameters, jabuticaba treatments did not restore the abundance of key bacterial genera reduced by DSS. In fact, in some cases, jabuticaba administration was associated with additional reductions in *Escherichia coli*, *Clostridium*, *Lactobacillus*, *Bifidobacterium*, and *Bacteroidetes*. These findings highlight an apparent paradox in the present study, as beneficial physiological outcomes in the host occurred despite the lack of microbiota restoration and, in some cases, a further decrease in specific bacterial populations. One possible explanation is that the reductions may represent a transient effect related to the high concentration of bioactive phenolic compounds present in jabuticaba [16,19]. Polyphenols are known to exert antimicrobial activity through mechanisms such as disruption of bacterial membranes, interference with microbial metabolism, and metal chelation. In acute exposure models, such as the short-duration in ovo DSS model used in this study, these antimicrobial properties may temporarily suppress the growth of both beneficial and opportunistic bacteria before microbial adaptation or ecological restructuring occurs [66,67,68,69].

Importantly, the reduction observed affected both traditionally beneficial and potentially opportunistic genera, suggesting that jabuticaba treatment modulated microbial abundance in a non-discriminatory manner under acute inflammatory conditions. Given the short duration of exposure and the acute nature of the in ovo DSS model, it is plausible that polyphenol–microbiota interactions occurred before adaptive microbial restructuring could take place [70,71]. Importantly, the persistence of intestinal and brain benefits in the absence of microbiota restoration suggests that jabuticaba’s primary effects in this model may be mediated through direct modulation of host signaling pathways rather than through major restructuring of the microbial ecosystem. Additionally, the microbiota analysis was based on the targeted qPCR quantification of a limited number of pre-selected genera rather than a comprehensive 16S rRNA sequencing approach. Therefore, the observed changes should be interpreted with caution, as they do not reflect the full microbial community structure or potential compensatory shifts in other bacterial taxa.

The intestinal improvements observed in the treated groups have direct implications for the liver, given the role of the gut–liver axis as a bidirectional route through which microbial mediators, cytokines, and endotoxins reach the liver, shaping inflammatory or metabolic responses [72]. Interestingly, in the present study, DSS did not increase hepatic NF-κB expression compared with the water group, suggesting that, unlike what is observed in murine models, the in ovo model does not develop classical hepatic inflammation [12,73,74,75]. However, the significant increase in iNOS expression and the presence of morphological hepatic alterations, such as reduced hepatocyte cytoplasm, increased fat accumulation, and greater inflammatory infiltrates, indicate the presence of oxidative stress and subclinical inflammation. This dissociation between iNOS and NF-κB has been described in situations where oxidative stress precedes or occurs independently of the activation of transcriptional inflammatory pathways, especially in models of acute intestinal injury, where barrier integrity is compromised but systemic inflammation is not fully established [76,77,78].

All three forms of jabuticaba effectively normalized iNOS expression and reduced hepatic antioxidant markers (CAT, SOD-1, and GSH-Px), indicating that supplementation reduced hepatic oxidative stress. The observed pattern suggests that jabuticaba, which is naturally rich in bioactive phenolic compounds with potent antioxidant and anti-inflammatory properties, not only minimizes nitric oxide and free radical production but also provides exogenous antioxidant support capable of neutralizing reactive oxygen and nitrogen species directly. This exogenous protection reduces the physiological demand for activating endogenous antioxidant defenses, particularly in the microencapsulated group, where the enhanced stability and biological efficacy of phenolics resulted in a markedly lower overall oxidative burden [20,22]. This was reflected in the histological analyses, which showed considerable reductions in hepatic fat deposition and inflammatory infiltrates across all treatments, along with recovery of hepatocyte cytoplasm. These findings are consistent with previous studies demonstrating the hepatoprotective capacity of jabuticaba through modulation of lipid, inflammatory, and oxidative pathways [21,79,80].

The results of the present study reinforce that the observed hepatic antioxidative protection did not occur in isolation but rather because of the intestinal barrier improvements promoted by jabuticaba. Thus, the beneficial effects can be interpreted within an integrated gut–liver axis framework in which restoration of epithelial integrity reduces the translocation of endotoxins and other pro-oxidative mediators to the liver, decreasing oxidative stress and improving hepatic function. The simultaneous modulation of intestinal NF-κB and hepatic iNOS suggests that jabuticaba phenolic compounds act both at the origin of the injury and on its systemic consequences.

Furthermore, increased intestinal permeability and inflammatory signaling can lead to the translocation of microbial products and cytokines into circulation, which in turn influence neural pathways and neurochemical balance [11,81,82]. In the present study, DSS exposure was associated with reduced brain gene expression of BDNF, despite the absence of increased COX-2 expression, indicating that the observed neural alterations were not driven by overt neuroinflammation. Instead, these findings suggest that intestinal injury and peripheral inflammatory signaling may impair neurotrophic support through gut–brain communication pathways independent of classical central inflammatory responses. Increased intestinal permeability and mucosal immune activation induced by DSS can promote the systemic release of cytokines and stress-related mediators that reach the brain via blood-borne or neural routes, thereby influencing neurotrophic signaling without necessarily activating COX-2-dependent inflammatory pathways in the brain [83,84,85].

BDNF is particularly sensitive to peripheral stressors and metabolic and inflammatory cues originating from the gut, especially during early developmental stages. Thus, the reduction in BDNF expression observed in this model likely reflects an indirect effect of intestinal inflammation on brain function, mediated by altered gut-derived signaling rather than local neuroinflammation. Given the central role of BDNF in neuronal plasticity, synaptic development, and stress resilience, early-life reductions in its expression may have lasting consequences for brain development and function [86,87,88].

Interestingly, jabuticaba treatments reversed DSS-induced reductions in BDNF gene expression, with the jabuticaba peel group exhibiting levels that exceeded those of the healthy control. This observation suggests a neurotrophic-supportive effect of jabuticaba that may be secondary to improved intestinal homeostasis or mediated by bioactive compounds capable of influencing brain signaling directly or indirectly [89,90]; however, this interpretation should be considered cautiously, as the present study evaluated only gene expression markers without complementary histological or functional assessments of neural tissue. In addition, all jabuticaba-treated groups showed increased dopamine-related expression and reduced COX-2 expression in the brain. Dopamine is a key neurotransmitter involved in motivation, reward, and cognitive processes, and its modulation by dietary bioactives has been increasingly linked to gut-derived signaling mechanisms [91,92]. Emerging evidence suggests that its modulation can also influence gut physiology via peripheral dopaminergic signaling. Enteric dopaminergic pathways have been shown to regulate intestinal motility, mucosal secretion, and epithelial integrity through dopamine receptors expressed along the gastrointestinal tract, independent of classical central inflammatory responses. Peripheral dopamine signaling may thus contribute to the maintenance of intestinal homeostasis and gut–brain communication via neural and circulatory routes [93,94,95].

Overall, this study demonstrates that jabuticaba-derived products, particularly microencapsulated peel formulations, mitigate DSS-induced intestinal inflammation, enhance barrier integrity, modulate the liver homeostasis, and modulate brain-related molecular markers associated with neurotrophic support and neurotransmitter signaling in an in ovo model. These findings suggest that jabuticaba-derived products may modulate molecular pathways involved in gut–liver–brain axis signaling during inflammatory challenges in this experimental model. However, the translational relevance of these effects to mammalian physiology and human health requires further investigation. Additionally, the experimental design included a relatively small sample size, which is partly inherent to the constraints of the in ovo model and hatching success rates. Consequently, the study should be considered exploratory, and further investigations with larger experimental groups are warranted.

Furthermore, it is worth mentioning that, although the *Gallus gallus* in ovo model provides a useful platform for studying early intestinal responses to dietary bioactive compounds, its translational relevance to human physiology must be interpreted with caution. This model offers important advantages, including controlled nutrient exposure, reduced environmental variability, and the possibility of investigating early host responses during intestinal development. However, significant differences exist between avian embryos and mammalian systems, particularly regarding immune system maturation, intestinal physiology, and the complexity and stability of the gut microbiota. Additionally, the short experimental window characteristic of the in ovo approach primarily reflects acute responses rather than long-term physiological adaptations. Therefore, the findings presented here should be considered as mechanistic and exploratory evidence supporting the biological activity of jabuticaba-derived compounds.

Although this study provides novel evidence regarding the protective effects of jabuticaba, certain limitations must be considered when interpreting the findings. First, the in ovo *Gallus gallus* model represents an early developmental system that allows the investigation of intestinal and systemic responses under control conditions; however, the findings should be interpreted cautiously when extrapolating to fully developed organisms or human physiology, and the results should be interpreted primarily as reflecting acute developmental responses rather than chronic inflammatory processes. In addition, the evaluation was performed at a single time point immediately after hatching, thus limiting the assessment of longer-term physiological adaptations and sustained effects of jabuticaba compounds.

Another limitation is that several outcomes were assessed primarily at the gene expression level, without complementary protein quantification or functional assays that could further confirm the biological mechanisms involved. Finally, although changes in selected cecal bacterial populations were evaluated, the study did not employ high-throughput microbiome sequencing approaches, which could provide a more comprehensive characterization of microbial community shifts associated with the treatments. Also, it is important to note that the microbial profile obtained in newly hatched chicks primarily reflects early colonization dynamics rather than a fully established physiological microbiota. Therefore, future studies using longer experimental periods, additional functional analyses, and more detailed microbiome profiling are warranted to further elucidate the mechanisms by which jabuticaba modulates the gut–liver–brain axis.

## 5. Conclusions

This study indicates that jabuticaba-derived products, including freeze-dried whole fruit, freeze-dried peel, and microencapsulated peel extract, were able to attenuate several DSS-induced alterations along the gut–liver–brain axis in an in ovo (*Gallus gallus*) model. All processing forms were associated with improvements in markers related to intestinal barrier integrity, including increased expression of mucin- and tight junction-related genes, higher goblet cell numbers, and reduced inflammatory signaling. Among the tested formulations, the microencapsulated peel tended to show more pronounced effects in some barrier-related parameters, suggesting that processing strategies may influence the biological activity of jabuticaba-derived compounds.

Beyond intestinal outcomes, DSS exposure was associated with signs of hepatic oxidative stress and changes in brain molecular markers. Jabuticaba treatments were associated with normalization of hepatic oxidative stress markers and modulation of genes related to neurotrophic signaling, including BDNF. However, these neural findings should be interpreted cautiously, as they were limited to gene expression analyses and were not supported by complementary functional or histological assessments. Similarly, although some changes in cecal bacterial populations were observed, the targeted microbiota analysis used in this study does not allow for a comprehensive evaluation of microbial community dynamics.

Overall, the findings suggest that jabuticaba-derived preparations may influence molecular pathways associated with intestinal barrier function and systemic responses in this experimental model. However, given the developmental nature of the in ovo system and the acute exposure conditions, the results should be interpreted as exploratory and mechanistic. Further studies in mammalian models, including detailed microbiome, metabolomic, and bioavailability analyses, are necessary to better understand the physiological relevance and translational implications of these observations.

## Figures and Tables

**Table 1:** Osmolarity of whole, peel, and microencapsulated jabuticaba at different concentrations.

Sample	% (*p*/*v*)	Osmolarity (mmol/kg)
**Whole** **jabuticaba**	1%	75
	5%	170
	10%	311
**Jabuticaba** **peel**	1%	75
	5%	164
	10%	320
**Jabuticaba** **microencapsulated**	1%	63
	5%	83
	10%	112

**Table 2:** Sequence of primers for the RT-qPCR analysis.

Genes	Oligonucleotide (5′-3′)	
	*Forward*	*Reverse*
***Inflammatory genes***		
NF-κB	F: 5′-TGT ACC TGC AGC CGG AAT G-3′	R: 5′-GGT AAT GGT TTA CGC GGA TGG-3′
IL-1β	F: 5′-CCC TCC TCC AGC CAG AAA G-3′	R: 5′-ACG TCG GCT TCC TCC A-3′
COX-2	F: 5′-TGT CCT TTC ACT GCT TTC CAT-3′	R: 5′-TTC CAT TGC TGT GTT TGA GGT-3′
***Oxidative stress genes***		
iNOS	F: 5′-GAG CAC TCA TGA CCC CAA AG-3′	R: 5′-GGG CCA GGT GCT CTT CTA TT-3′
CAT	F: 5′-ACT GCA AGG CGA AAG TGT TT-3′	R: 5′-GGC TAT GGA TGA AGG ATG GA-3′
SOD-1	F: 5′-TGC TTG CCT TCA GGA TTA AAG TGA G-3′	R: TTG TCT GAT GGA GAT CAT GGC TTC
GSH-Px	F: 5′-TCA CCA TGT TCG AGA AGT GC-3′	R: 5′-ATG TAC TGC GGG TTG GTC AT-3′
***Intestinal health genes***		
MUC-2	F: 5′-CCT GCT GCA AGG AAG TAG AA-3′	R: 5′-GGA AGA TCA GAG TGG TGC ATA G-3′
JAM-2	F: 5′-AAG GAT TCT GGG ACC TAC CG-3′	R: 5′-GTT CCC GTC ATT GCA GAG TT-3′
CLDN-1	F: 5′-CTT CAT CAT TGC AGG TCT GTC AG-3′	R: 5′-AAA TCT GGT GTT AAC GGG TGT G-3′
ZO-2	F: 5’-GAA AGC AGA CCC TGC TCA AC-3’	R: 5’-TGG ATG AAT GCA AAT CCA GA-3’
***Brain health genes***		
Dopamine	F: 5’-TCG TCC TCA TCC TGC TCA TC-3’	R: 5’-GTG GGC ATA AGG GTG GTA CT-3’
BDNF	F: 5’-ACA AGC GAG TGG GTA ACA GC-3’	R: 5’-CTT GGG GTT GCA TTT GGT CTC-3’
***Endogenous gene***		
β-actin	F: 5’-ATG GCT CCG GTA TGT GCA AG-3’	R: 5’-CAA CCA TCA CAC CCT GAT GTC-3’

NF-κB, nuclear factor kappa B; IL-1β, interleukin-1β; MUC-2: Mucin 2; JAM-2: Junctional adhesion molecule 2; CLDN-1: Claudin 1; ZO-2, zonula occludens-2; iNOS, inducible nitric oxide synthase; CAT, catalase; SOD-1, superoxide dismutase 1; GSH-Px, glutathione peroxidase; COX-2, cyclooxygenase-2; BDNF, brain-derived neurotrophic factor.

**Table 3:** Phenolic content, anthocyanin concentration, and antioxidant capacity of jabuticaba’s solutions.

Analysis	Concentration		
	WJ	JP	JM
Total phenolic content (mg GAE/mL)	1.68 ± 0.04	3.19 ± 0.22	0.52 ± 0.00
Monomeric anthocyanins (mg C3G equivalent/mL)	0.29 ± 0.02	0.73 ± 0.01	0.09 ± 0.00
DPPH (µM Trolox/mL)	160.74 ± 11.54	179.63 ± 7.63	45.15 ± 2.03
ABTS (µmol TE/mL)	19.93 ± 0.10	21.38 ± 1.88	4.27 ± 0.60
FRAP (µmol TE/mL)	36.53 ± 3.57	48.17 ± 1.13	6.04 ± 0.70

WJ, whole jabuticaba; JP, jabuticaba peel; JM, jabuticaba microencapsulated. GAE, gallic acid equivalent; C3G, cyanidin-3-glucoside; DPPH, 1,1-diphenyl-2-picrylhydrazyl; ABTS, 2,2′-azino-bis-3-ethylbenzthiazoline-6-sulphonic acid; FRAP, ferric-reducing antioxidant power; TE, Trolox equivalent.

**Table 4:** Histomorphometry analysis in duodenal of chicks.

Variables	Groups				
	Water	DSS	WJ + DSS	JP + DSS	JM + DSS
Longitudinal thickness (µm)	13.79 ± 2.81 ^ab^	9.26 ± 1.51 ^c^	10.71 ± 0.88 ^bc^	14.64 ± 2.06 ^a^*	10.11 ± 1.86 ^c^
Circular thickness (µm)	38.96 ± 4.98 ^a^	35.68 ± 5.94 ^a^	34.15 ± 2.26 ^a^	37.71 ± 6.44 ^a^	35.72 ± 6.30 ^a^
Villi length (µm)	189.20 ± 12.32 ^a^	155.90 ± 19.74 ^a^	182.20 ± 23.82 ^a^	156.60 ± 20.46 ^a^	166.00 ± 34.62 ^a^
Villi width (µm)	38.32 ± 4.12 ^a^	41.59 ± 4.98 ^a^	46.11 ± 4.96 ^a^	36.62 ± 3.50 ^a^	38.36 ± 7.39 ^a^
Villi surface (µm^2^)	22,213.85 ± 2257.12 ^c^	19,560.12 ± 3404.22 ^c^	49,692.95 ± 2544.92 ^a^*	35,529.63 ± 5281.10 ^b^*	40,214.88 ± 5189.57 ^b^*
Crypt length (µm)	30.26 ± 3.92 ^bc^	36.68 ± 2.30 ^a^	33.66 ± 3.63 ^ab^	24.85 ± 1.85 ^c^*	29.57 ± 3.77 ^bc^*
Crypt depth (µm)	18.81 ± 1.74 ^a^	20.58 ± 3.03 ^a^	17.34 ± 0.97 ^a^	16.49 ± 3.04 ^a^	18.16 ± 3.58 ^a^
Goblet cell per villi	23.13 ± 4.05 ^bc^	19.51 ± 0.57 ^c^	29.03 ± 4.31 ^a^*	24.41 ± 1.42 ^ac^*	25.07 ± 2.31 ^ab^*
Goblet cell per crypt	4.77 ± 1.21 ^c^	5.25 ± 1.01 ^c^	8.25 ± 0.65 ^a^*	6.03 ± 0.44 ^bc^	7.33 ± 0.74 ^ab^*
Goblet cell area (µm^2^)	11.31 ± 1.10 ^bc^	11.48 ± 1.79 ^bc^	11.51 ± 1.52 ^bc^	10.68 ± 2.03 ^c^	13.62 ± 0.36 ^ab^*

Water, 18 MΩ H_2_O; DSS, dextran sulfate sodium 1.5%; WJ + DSS, whole jabuticaba (5%) + DSS (1.5%); JP + DSS, jabuticaba peel (5%) + DSS (1.5%); JM + DSS, jabuticaba microencapsulated (5%) + DSS (1.5%). n = 5/group. * Indicates significant differences between the DSS groups (DSS × WJ + DSS; DSS × JP + DSS; DSS × JM + DSS), according to the *t*-test (*p* < 0.05). Different lowercase letters (a–c) indicate significant differences within groups, according to ANOVA, followed by Tukey’s test, at 5% probability. Data expressed as mean ± standard deviation.

**Table 5:** Morphometric outcomes from colon histology.

Variables	Groups				
	Water	DSS	WJ + DSS	JP + DSS	JM + DSS
Longitudinal thickness (µm)	24.71 ± 2.96 ^b^	44.76 ± 3.27 ^a^	25.96 ± 2.79 ^b^*	16.33 ± 2.33 ^c^*	18.18 ± 2.18 ^c^*
Circular thickness (µm)	68.06 ± 3.69 ^b^	79.56 ± 3.27 ^a^	81.25 ± 3.99 ^a^	57.21 ± 3.65 ^c^*	58.13 ± 3.32 ^c^*
Crypt depth (µm)	81.22 ± 4.15 ^b^	66.76 ± 2.12 ^c^	92.73 ± 3.35 ^a^*	80.47 ± 6.56 ^b^*	84.69 ± 5.66 ^ab^*
Crypt width (µm)	45.18 ± 3.13 ^bc^	42.00 ± 2.87 ^c^	51.90 ± 2.99 ^ab^*	43.39 ± 4.90 ^c^	48.24 ± 3.56 ^bc^*
Goblet cell per crypt	37.96 ± 1.51 ^bc^	33.71 ± 2.81 ^c^	48.46 ± 3.68 ^a^*	42.41 ± 4.45 ^ab^*	40.99 ± 2.91 ^b^*
Goblet cell area (µm^2^)	13.22 ± 1.23 ^cd^	11.89 ± 1.00 ^d^	21.23 ± 2.76 ^a^*	16.19 ± 1.79 ^bc^*	17.66 ± 2.02 ^b^*

Water, 18 MΩ H_2_O; DSS, dextran sulfate sodium 1.5%; WJ + DSS, whole jabuticaba (5%) + DSS (1.5%); JP + DSS, jabuticaba peel (5%) + DSS (1.5%); JM + DSS, jabuticaba microencapsulated (5%) + DSS (1.5%). n = 5/group. * Indicates significant differences between the DSS groups (DSS × WJ + DSS; DSS × JP + DSS; DSS × JM + DSS), according to the *t*-test (*p* < 0.05). Different lowercase letters (a–d) indicate significant differences within groups, according to ANOVA, followed by Tukey’s test, at 5% probability. Data expressed as mean ± standard deviation.

**Table 6:** Histomorphometry point-count analysis of liver tissue.

Variables	Groups				
	Water	DSS	WJ + DSS	JP + DSS	JM + DSS
Liver nucleus	28.50 ± 2.83 ^b^	26.70 ± 1.52 ^b^	27.08 ± 0.93 ^b^	24.58 ± 2.69 ^b^	32.79 ± 2.87 ^a^*
Cytoplasm	136.40 ± 3.02 ^c^	113.40 ± 5.39 ^d^	155.40 ± 4.59 ^b^*	165.00 ± 4.04 ^a^*	148.50 ± 2.45 ^b^*
Fat globule	62.10 ± 1.88 ^b^	86.45 ± 3.89 ^a^	49.25 ± 4.68 ^c^*	47.79 ± 3.76 ^c^*	45.67 ± 3.29 ^c^*
Inflammatory infiltrates	1.56 ± 0.31 ^a^	1.96 ± 0.25 ^a^	0.67 ± 0.30 ^b^*	0.54 ± 0.19 ^b^*	0.67 ± 0.13 ^b^*

Water, 18 MΩ H_2_O; DSS, dextran sulfate sodium 1.5%; WJ + DSS, whole jabuticaba (5%) + DSS (1.5%); JP + DSS, jabuticaba peel (5%) + DSS (1.5%); JM + DSS, jabuticaba microencapsulated (5%) + DSS (1.5%). n = 5/group. * Indicates significant differences between the DSS groups (DSS × WJ + DSS; DSS × JP + DSS; DSS × JM + DSS), according to the *t*-test (*p* < 0.05). Different lowercase letters (a–d) indicate significant differences within groups, according to ANOVA, followed by Tukey’s test, at 5% probability. Data expressed as mean ± standard deviation.

## Data Availability

The original contributions presented in this study are included in the article. Further inquiries can be directed to the corresponding author.
